# Context-specific complementary feeding recommendations developed using Optifood could improve the diets of breast-fed infants and young children from diverse livelihood groups in northern Kenya

**DOI:** 10.1017/S1368980016003116

**Published:** 2016-12-05

**Authors:** Marieke Vossenaar, Frances A Knight, Alison Tumilowicz, Christine Hotz, Peter Chege, Elaine L Ferguson

**Affiliations:** 1 Global Alliance for Improved Nutrition (GAIN), Rue de Vermont 37–39, CH-1202 Geneva, Switzerland; 2 Kenyatta University, Nairobi, Kenya; 3 Faculty of Epidemiology and Population Health, London School of Hygiene & Tropical Medicine, London, UK

**Keywords:** Optifood, Linear programming, Infant and young child feeding, Complementary feedingrecommendations, Kenya

## Abstract

**Objective:**

To formulate age- and context-specific complementary feeding recommendations (CFR) for infants and young children (IYC) and to compare the potential of filling population-level nutrient gaps using common sets of CFR across age groups.

**Design:**

Linear programming was used to develop CFR using locally available and acceptable foods based on livelihood- and age-group-specific dietary patterns observed through 24 h dietary recalls. Within each livelihood group, the nutrient potential of age-group-specific *v*. consolidated CFR across the three age groups was tested.

**Setting:**

Three food-insecure counties in northern Kenya; namely, settled communities from Isiolo (*n* 300), pastoralist communities from Marsabit (*n* 283) and agro-pastoralist communities from Turkana (*n* 299).

**Subjects:**

Breast-fed IYC aged 6–23 months (*n* 882).

**Results:**

Age-specific CFR could achieve adequacy for seven to nine of eleven modelled micronutrients, except among 12–23-month-old children in agro-pastoralist communities. Contribution of Fe, Zn and niacin remained low for most groups, and thiamin, vitamin B_6_ and folate for some groups. Age-group-consolidated CFR could not reach the same level of nutrient adequacy as age-specific sets among the settled and pastoralist communities.

**Conclusions:**

Context- and age-specific CFR could ensure adequate levels of more modelled nutrients among settled and pastoralist IYC than among agro-pastoralist communities where use of nutrient-dense foods was limited. Adequacy of all eleven modelled micronutrients was not achievable and additional approaches to ensure adequate diets are required. Consolidated messages should be easier to implement as part of a behaviour change strategy; however, they would likely not achieve the same improvements in population-level dietary adequacy as age-specific CFR.

In Kenya, the arid and semi-arid land region experiences high rates of poverty, poor access to basic services and malnutrition^(^ ^1^ ^–^ ^3^ ^)^. This region is also one of the most vulnerable in the world to environmental shocks like drought and climate change^(^ ^2^ ^)^. Pastoralism is one of the few livelihoods appropriate for this environment because people can move livestock according to the ever-changing availability of water and pasture^(^ ^4^ ^)^. However, conflicts associated with resource scarcity are increasingly common and many are no longer able to make a living from pastoralism. As a result, pastoralists may transition to a sedentary existence, settled either in small rural villages or larger towns. However, moving from a pastoralist to an agro-pastoralist or settled livelihood status often further impoverishes families and many survive on a combination of food aid, small income earned through production of firewood or charcoal, gathering wild foods, fishing or begging^(^ ^4^ ^,^ ^5^ ^)^.

The US Agency for International Development (USAID)-funded ‘Resilience and Economic Growth in Arid Lands – Improving Resilience’ (REGAL-IR) project works in Garissa, Isiolo, Marsabit, Turkana and Wajir counties to build the capacity of communities to cope with and rebound from shocks such as recurring drought. REGAL-IR interventions support local structures to improve the social, economic and environmental conditions that contribute to nutritionally inadequate infant and young child (IYC) diets and high rates of child stunting^(^ ^6^ ^,^ ^7^ ^)^. At the same time, the project aims to improve nutrition outcomes through complementary feeding behaviour change interventions. The Global Alliance for Improved Nutrition (GAIN) was requested by USAID and the REGAL-IR project implementer, African Development Solutions (Adeso), to identify dietary strategies that would contribute to improving the nutrient adequacy of IYC diets. Based on this directive, we identified two main objectives that needed to be addressed: (i) identifying nutrient requirements that are currently not being met in IYC diets, together with the potential of local foods to improve the nutrient quality of their diets; and (ii) identifying cultural-ecological determinants of IYC feeding that serve as facilitators or barriers for the implementation of dietary improvement strategies. Examination of these two challenges required different study designs and methods. Therefore, we created two streams of inquiry: (i) nutrient gap analysis and development of complementary feeding recommendations (CFR); and (ii) examination of socio-ecological determinants using the GAIN-initiated Focused Ethnographic Study (FES) of the determinants of IYC feeding. The present paper reports the results of the first stream of inquiry aimed at developing CFR; the results of the FES study are reported elsewhere^(^ ^8^ ^)^.

The ‘1000 d’ from conception to age 2 years is widely recognized as a window of opportunity for the prevention of malnutrition and its short- and long-term consequences^(^ ^9^ ^,^ ^10^ ^)^. The longest stage in this window is the period of complementary feeding, which ideally starts at 6 months of age and continues up to 2 years of age, in which IYC move from being exclusively breast-fed to receiving nutrients from a range of foods in addition to breast milk. Achieving nutrient adequacy during this period is challenging as IYC eat in small amounts and nutrient adequacy can only be achieved through the consumption of nutrient-dense foods^(^ ^11^ ^,^ ^12^ ^)^. Developing CFR to improve nutrient adequacy is further complicated by rapid changes in consistency, amount and variety of complementary foods of the IYC diet as an infant ages.

While general IYC feeding guidelines have been developed for international use^(^ ^13^ ^)^, it is necessary to adapt these to be locally relevant. To facilitate the development of context-specific and realistic CFR for adoption by caregivers, a new software, Optifood, which uses linear programming analysis, was recently developed^(^ ^14^ ^)^. Optifood provides information on the best combinations of local foods to optimize nutrient intakes and can objectively indicate the extent to which these can supply nutritionally adequate diets for an entire population. The strength of Optifood is the capacity to identify CFR that are specific to the local context in terms of actual dietary practices, likely gaps between intakes and requirements for specific nutrients, and local food availability, which are typically defined by agro-ecologies and demographic and cultural influences. However, as the analyses used to identify CFR are based on 24 h dietary recall data representing food intakes specific to certain age groups (i.e. 6–8, 9–11 and 12–23 months), and as the dietary patterns across these age groups can vary, the Optifood-derived CFR can also differ substantially between age groups within a population area. Therefore, once CFR have been objectively selected for each age group within a population area, consideration should be given to adapting the CFR to foods and messages that are consistent, or consolidated, and progressive across age groups.

The analysis presented herein includes nine target groups defined on the basis of three age ranges (6–8 months; 9–11 months; 12–23 months) and three different livelihood groups (settled; pastoralist; agro-pastoralist) found in the REGAL-IR project implementation area. The paper first presents how Optifood was used for each of the nine target groups to identify nutrients for which adequacy cannot be reached or would be difficult to reach using local foods as they are usually consumed, and develops sets of CFR to best ensure nutrient intake adequacy for the population. Following this, based on the results from individual age- and livelihood-specific groups, the paper describes the process of consolidating CFR across age groups within each livelihood group and compares the nutrient adequacy of age-specific *v*. consolidated CFR.

## Methods

### Study design

Cross-sectional dietary and anthropometric data were collected in November and December 2013 from 6–23-month-old breast-fed IYC living in selected arid and semi-arid, rural communities in northern Kenya. The season of the survey is considered a season of ‘plenty’ because it corresponds to the period when the short rains are expected, pasture for livestock is usually available and when home-produced food is more available than at other times of the year. The dietary survey was part of a landscape analysis intended to inform the selection of interventions –specifically, behaviour change communication interventions – that could improve nutrient adequacy of IYC diets as part of the REGAL-IR project. In the present study, these data were analysed using linear programming analyses, in the Optifood software, to develop context-specific CFR.

### Participants and sampling

Three main livelihood groups from three food-insecure counties in northern Kenya were selected to represent distinct agro-ecological zones; namely, settled communities from Isiolo, pastoralist communities from Marsabit and agro-pastoralist communities from Turkana. All counties represent sparsely populated (<15 people/km^2^), arid areas of the eastern (Isiolo and Marsabit) and Rift Valley (Turkana) regions^(^ ^3^ ^)^. These areas are characterized by high under-5 stunting rates (30.9 % in the Rift Valley and 32.8 % in the Eastern region)^(^ ^1^ ^)^ and large numbers of impoverished households^(^ ^2^ ^,^ ^3^ ^)^.

Communities to be surveyed were selected on the basis of two criteria: (i) the REGAL-IR project was being implemented; and (ii) the majority of the population in the community represented the selected livelihood group for that county. The survey aimed to collect information from ninety-five breast-fed children from each of the three target age groups, i.e. 6–8-, 9–11- and 12–23-month-old children, in each of the three livelihood groups, based on the number of respondents needed to estimate population mean food serving sizes for commonly consumed foods to within±10 % (95 % CI) assuming an sd of 50 % of the mean serving sizes. Non-breast-fed IYC were not included in this analysis because the sample size of children that were not receiving breast milk was too small to analyse as a separate Optifood target group. A census was conducted in the selected sub-locations to identify all households with children aged 6–23 months and their breast-feeding status. For children aged 6–11 months, all breast-feeding children were invited to participate; whereas for children aged 12–23 months, a sample of breast-feeding children was randomly selected from each of the sub-location census lists.

### Data collection

Quantitative dietary data were collected for IYC using a multiple-pass 24 h dietary recall adapted from Gibson and Ferguson^(^ ^15^ ^)^. In the first pass, the primary caregiver was asked to sequentially recall all foods and drinks consumed by their child during the previous day, after which details such as brand names for commercially processed foods and portion sizes were recalled and recorded. Standard recipe data were collected from each livelihood group for recipes commonly used in IYC feeding^(^ ^15^ ^)^; for recipes reported to be consumed that were different from standard recipes, information on the ingredients, their amounts and the total cooked weight of the dish was collected during the 24 h recall interview. Portion sizes were estimated by asking caregivers to show the amount served, and the amount left over after feeding, using real foods; these amounts were weighed on dietary scales, recorded, and the amount consumed by the child was calculated by difference. In addition, a separate 24 h dietary recall was collected for the primary caregiver following similar methodology.

IYC anthropometric measurements were made in triplicate, by trained enumerators. Weight was measured to the nearest 100 g with an electronic scale (Seca model 770; Seca, Hamburg, Germany) and length was measured to the nearest 0.1 cm with a wooden measuring board. General household data relating to sociodemographic characteristics and food/hunger were collected using an interviewer-administered structured questionnaire.

Data were collected by thoroughly trained and supervised field staff. Dietary and anthropometric data were double-entered and underwent rigorous cleaning prior to analysis.

### Data analysis

Maternal diet quality was assessed using the Women’s Dietary Diversity Score, an indicator derived from a single 24 h dietary recall and calculated as the sum of food groups consumed (from a maximum of ten food groups)^(^ ^16^ ^)^, and the Household Hunger Scale was used to assess the food security situation^(^ ^17^ ^)^.

All IYC data were prepared and analysed by strata defined by age group (6–8 months, 9–11 months and 12–23 months) and livelihood group (settled, pastoralist and agro-pastoralist communities). *Z*-scores for IYC length-for-age and weight-for-length were estimated using WHO Anthro software version 3.1 based on the 2006 WHO Child Growth Standards^(^ ^18^ ^)^; and children with *Z*-scores<–2 were classified as stunted or wasted, respectively.

#### Linear programming analysis using Optifood

Optifood version 4.0.9.0 was used to generate a series of optimized modelled 7d diets that identified: (i) problem nutrients (i.e. nutrients likely to remain low in IYC diets based on local food sources, as consumed); (ii) the best available food sources to fill nutrient gaps; and (iii) alternative CFR for 7d diets that would improve dietary adequacy for eleven nutrients (Ca, Fe, Zn, riboflavin, niacin, thiamin, folate and vitamins A, B_6_, B_12_ and C). These alternative CFR were then tested, in Optifood, to select the best sets of CFR, using criteria based on predicted population-level nutrient adequacy and minimum diet cost. The process of analysing dietary data and developing CFR with Optifood has been described in detail elsewhere^(^ ^14^ ^,^ ^19^ ^–^ ^21^ ^)^. In the present study, all analyses were done in Modules I to III of Optifood for each of the nine target groups. Specific parameters used in these analyses are described below.

#### Setting model parameters in Optifood

The model parameters for Optifood were generated using the dietary survey data, which were entered and processed using CS Dietary software version 1.1 and a Microsoft^®^ Access 2010 program developed to support Optifood (D Wiesmann and E Ferguson, unpublished results). The constraints used in all analyses to ensure realistic modelled diets were: (i) the energy (kilocalorie) content of each modelled diet, which was equal to the average energy requirement for the target group; (ii) the minimum and maximum (generally corresponding to the 10th and 90th percentiles, respectively) number of servings from food groups and food subgroups per week; and (iii) the minimum and maximum grams of each individual food item per week. These 7d parameters were estimated using individual 24 h dietary recalls multiplied by 7 to simulate weekly food intakes. The serving sizes, for all food items (grams per meal), were determined as the median portion size for consumers in each target group. All food items reported in the 24 h dietary recalls, for each target group, were included in the analyses with the exception of condiments (spices, salt), foods of no nutritional value (tea), therapeutic foods (i.e. Plumpy’Nut^®^) and foods deemed uncommon by local informants.

As the quantity of breast milk consumed was not assessed in the dietary survey, daily intakes were estimated based on an assumption that, on average, breast milk contributes 67, 55 and 39 % of the median energy requirements for children aged 6–8, 9–11 and 12–23 months, respectively^(^ ^22^ ^)^. Assuming breast milk energy content of 2·76 kJ/g (0·66 kcal/g)^(^ ^22^ ^)^, the daily intake of breast milk was estimated as 570, 520 and 420 g for children aged 6–8, 9–11 and 12–23 months, respectively. The nutrient composition of breast milk for ‘developing countries’ was assumed^(^ ^22^ ^)^.

The primary source of food composition data for all individual foods was the US Department of Agriculture’s National Nutrient Database, Release 23^(^ ^23^ ^,^ ^24^ ^)^. Energy and protein requirements for each target group were estimated using the relevant WHO/FAO algorithms and the average body weight by age group taken from the survey data (shown in Table 1)^(^ ^25^ ^,^ ^26^ ^)^. The 2004 FAO/WHO Recommended Nutrient Intakes (RNI) were used for all target groups^(^ ^27^ ^)^. Given that animal-source food consumption was meagre, assumptions for the dietary bioavailability of Zn and Fe were set at 15 and 5 %, respectively. In addition to energy, protein and fat, a total of eleven micronutrients (listed above) were examined in the Optifood analysis.

#### Identifying problem nutrients

Two classifications of problem nutrients, ‘absolute’ and ‘partial’, were identified through Optifood analysis. Absolute problem nutrients were defined as those nutrients that were <100 % of the RNI in the Module II nutritionally best diet and <100 % of the RNI in the Module III maximized diets modelled without CFR constraints. Partial problem nutrients were defined as those nutrients that were <100 % of the RNI in the Module II nutritionally best diet, but ≥100 % of the RNI in the Module III maximized diets modelled without CFR constraints. This meant that the RNI for that nutrient, in at least one modelled diet, could be achieved using local foods as consumed, but at the expense of meeting the RNI for other nutrients.

#### Selection of complementary feeding recommendations

Individual CFR selected for screening in Module III were identified in two ways: (i) food groups that had a higher number of servings of foods in the Module II nutritionally best diet than the median observed for those food groups (i.e. food group patterns that have nutritional benefits); and (ii) individual food items that provided≥5 % of the nutrient content for at least five micronutrients in the Module II nutritionally best diet (i.e. the best food sources of nutrients), generally expressed at their food subgroup level.

#### Testing complementary feeding recommendations

The individual CFR were first screened in Module III (minimized 7d diets) to select a subset of individual CFR for a systematic analysis. To select this subset, the Module III minimized nutrient values of the individual CFR were compared to identify CFR that, when combined, would likely provide the highest levels of all eleven micronutrients modelled. In the systematic analysis, all possible combinations of these selected CFR were tested. In these series of analyses, population-level dietary adequacy for a nutrient was defined as a minimized 7d diet value≥65 % of its RNI. Thus, a cut-off of 65 % of the RNI, for the simulated diet with the lowest nutrient content, for each nutrient, was predicted to ensure nutrient adequacy for nearly the entire population.

#### Consolidation of complementary feeding recommendations

Following the selection of the best set of CFR for each livelihood- and age-specific target group, the CFR were compared and consolidated across age groups within each livelihood group with the aim to develop common sets of CFR for each livelihood group. Given the heterogeneity of the study populations, no attempt was made to consolidate CFR across different livelihood groups. The approach used to consolidate CFR across age groups within each livelihood group was to select CFR that were recommended for at least two of the three age groups (i.e. food groups or subgroups with the greatest nutrient contribution). In practice, a CFR proposed for a single age group was eliminated, whereas a CFR proposed for at least two age groups was added to the remaining age groups even if it had not previously been identified as a potential CFR in that target group. Once a consolidated set of CFR was selected, an analysis was carried out to test the percentage of the RNI of modelled nutrients achievable for each target group if consolidated CFR rather than age-specific CFR were used. Age-specific median portion sizes and frequencies of intake were maintained.

## Results

### Survey results

Pastoralist households sampled in Marsabit were predominantly from the Gabra ethnic group (Table 1), while the settled households from Isiolo and the agro-pastoralist households from Turkana were ethnically similar (both from the Turkana ethnic group). Among the settled and pastoralist communities foods were purchased or produced at home in similar proportions, whereas agro-pastoralist communities relied mostly on home production (87 %). The most common livestock owned by households across the three livelihood groups were goats, sheep and cattle. Camel ownership was common among pastoralist Marsabit communities (84 % of households), but uncommon among settled Isiolo and agro-pastoralist Turkana communities. Many households in settled and agro-pastoralist communities (48 and 64 % of households, respectively) owned chickens, compared with only a few (8 %) households in pastoralist Marsabit communities.

**Table 1:** Selected sociodemographic characteristic of breast-fed infants and young children, households and caregivers across three livelihood groups in northern Kenya, November–December 2013

	Settled communities (*n* 300)		Pastoralist communities (*n* 283)		Agro-pastoralist communities (*n* 299)	
Household and caregiver characteristics						
Tribe/ethnicity (% yes)						
Turkana	85		0		100	
Gabra	0		97		0	
Main source of food during plentiful season (%)						
Household food production	52		40		87	
Purchased	47		57		10	
Household Hunger Score* categories (%)						
Little or no hunger in the household (0–1)	24		56		6	
Moderate hunger in the household (2–3)	71		38		88	
Severe hunger in the household (4–6)	5		7		6	
Household participated in a food programme within the last year (% yes)	21		33		27	
	Mean	sd	Mean	sd	Mean	sd
Women’s Dietary Diversity Score† (no. of food groups)	3.4	1.5	3.3	0.9	2.7	1.2
Schooling of caregiver (%)						
Never attended	63		81		78	
Primary, incomplete	19		15		17	
Primary, completed	11		2		2	
Child characteristics						
No. of children surveyed						
6–8 months	91		68		69	
9–11 months	104		76		134	
12–23 months	105		139		96	
Stunting prevalence‡ (%)						
6–8 months	9.8		10.3		7.5	
9–11 months	11.7		6.5		9.6	
12–23 months	24.8		17.3		18.8	
Wasting prevalence§ (%)						
6–8 months	1.1		10.3		19.4	
9–11 months	11.7		16.9		25.2	
12–23 months	10.5		19.4		21.9	
	Median	25–75th percentile	Median	25–75th percentile	Median	25–75th percentile
Body weight (kg)						
6–8 months	7.5	7.1–8.1	7.6	6.7–8.2	7.3	6.8–8.3
9–11 months	8.2	7.4–8.7	8.1	7.7–8.7	7.8	7.3–8.6
12–23 months	9.0	8.3–9.7	8.8	8.1–9.7	8.9	8.1–9.9
Energy intake from complementary foods|| (kJ)						
6–8 months	1833	1314–2803	1623	1213–2314	1084	778–1439
9–11 months	2238	1531–3222	2050	1448–2561	1469	862–2008
12–23 months	2791	1962–3724	2531	1816–3213	1795	1029–2745

* The Household Hunger Score is used to assess the food security situation^(^ ^17^ ^)^.

† The Women’s Dietary Diversity Score is based on ten food groups; women consuming foods from five or more food groups have a greater likelihood of meeting their micronutrient needs than women consuming foods from fewer food groups^(^ ^42^ ^)^.

‡ Stunting is defined by a length-for-age<−2 sd from the median of the 2006 WHO Child Growth Standards^(^ ^18^ ^)^.

§ Wasting is defined by a weight-for-length<−2 sd from the median of the 2006 WHO Child Growth Standards^(^ ^18^ ^)^.

|| Based on a single 24 h dietary recall.

Among these northern Kenyan populations, a large proportion of households reportedly experienced hunger in the last month and dietary diversity for women was low (Table 1). These data suggest that Turkana agro-pastoralists suffered the greatest limitations in terms of the severity and prevalence of household hunger and limited dietary diversity. Between one-fifth and one-third of the population reported participating in a food programme within the last year (Table 1).

Although a significant percentage of children surveyed experienced growth faltering (Table 1), the prevalence of stunting in all three livelihood groups was less than it was in the national estimates (26.5 and 35.5 % for children aged 12–17 and 18–23 months, respectively)^(^ ^1^ ^)^. This difference may be related to very different growth patterns among pastoralist populations, whereby pastoral children have been found to grow up thinner but taller than children of agriculturalists^(^ ^28^ ^)^. It is also notable that a higher percentage of children aged 12–23 months were stunted (length-for-age *Z*-score<−2) among settled households in Isiolo than among the pastoralist communities. This finding is similar to other studies in the region that have found that when pastoralist populations settle the prevalence of stunting increases and the prevalence of wasting decreases^(^ ^5^ ^)^. The prevalence of wasting was high based on the WHO classification of severity of wasting: 11, 19 and 22 % of children aged 12–23 months were wasted (weight-for-length *Z*-score<−2) in Isiolo, Marsabit and Turkana, respectively.

Energy contribution from complementary foods increased with age, being highest in the settled communities and lowest in the agro-pastoralist communities. Estimated intakes ranged between 1084 kJ (259 kcal) for 6–8-month-old infants from the agro-pastoralist communities and 2791 kJ (667 kcal) for 12–23-month-old children from the settled communities. Across the three livelihood groups surveyed, the reported diets were based almost exclusively on four food groups, namely dairy, grain products, added fats and added sugar. These contributed a high proportion of daily energy intakes from the complementary feeding diets (82 % in settled, 89 % in pastoralist and 84 % in agro-pastoralist communities). Dairy (animal milk) was of greatest nutritional importance among pastoralist communities (46 % of energy intake from the complementary diet), of some importance among settled populations (28 % of energy intake) and of least relative importance among severely food-insecure, agro-pastoralist communities (8 % of energy intake). The dairy and grain foods consumed across livelihood groups were distinct. Camels’ milk was predominant among the pastoralists, goats’ and cows’ milk were most common among the settled populations, and goats’ milk and milk powder were consumed among agro-pastoralists. Rice and commercially packaged maize flour were the primary grain foods among settled and pastoralist communities, whereas among agro-pastoralists, local, low extraction maize flour was consumed. Additionally, potatoes and beans together contributed to energy intake (12 % in settled, 8 % in pastoralist and 11 % in agro-pastoralist communities). Vegetable consumption consisted almost entirely of small amounts of tomato and onion added to recipes, with the exception of the common consumption of amaranth leaves among settled communities. Fruit and non-dairy animal-source foods (i.e. meat, fish, poultry and eggs) were almost completely absent from reported diets.

### Linear programming analyses results

In the final food lists modelled, target groups from settled communities had a higher number and variety of foods available for modelling (*n* 57) than pastoralist (*n* 35) and agro-pastoralist (*n* 34) communities, indicative of a lack of variety and availability of food in these latter two livelihood groups.

#### Problem nutrients

A number of problem nutrients were identified for each target group (Table 2). Target groups in agro-pastoralist communities had the highest number of problem nutrients (five to seven of eleven modelled micronutrients), followed by pastoralist (three to five) and settled communities (two to four). Zn and Fe were consistently well below their RNI across all modelled diets (as low as 15 % of the requirement for Fe and 25 % for Zn in the Module II nutritionally best diet) and were classified as absolute problem nutrients, meaning that their RNI could not be achieved using the foods as modelled. Niacin was a problem nutrient in all but one target group; however, niacin might not be a problem nutrient when the contribution of tryptophan to niacin intakes is taken into account.

**Table 2:** Absolute* and partial† problem nutrients identified for each targeted age group of breast-fed infants and young children across three livelihood groups in northern Kenya, November–December 2013

	Settled communities (*n* 300)			Pastoralist communities (*n* 283)			Agro-pastoralist communities (*n* 299)		
	6–8-month-old infants	9–11-month-old infants	12–23-month-old children	6–8-month-old infants	9–11-month-old infants	12–23-month-old children	6–8-month-old infants	9–11-month-old infants	12–23-month-old children
Ca	Par†						Par†	Par†	Par†
Thiamin							Par†	Par†	Par†
Niacin	Par†		Par†	Par†	Par†	Par†	Par†	Par†	Abs*
Vitamin B_6_						Par†			Par†
Folate				Par†		Par†			Par†
Fe	Abs*	Abs*		Abs*	Abs*	Par†	Abs*	Abs*	Abs*
Zn	Abs*	Abs*	Abs*	Abs*	Abs*	Abs*	Abs*	Abs*	Abs*
No. of absolute problem nutrients	2	2	1	2	2	1	2	2	3
No. of partial problem nutrients	2	0	1	2	1	4	3	3	4

Abs, absolute problem nutrient; Par, partial problem nutrient.

* An ‘absolute’ problem nutrient is defined as a nutrient whose requirement (i.e. 100 % of the Recommended Nutrient Intake (RNI)) is impossible to meet using local foods within the model constraints for frequency and portion size (identified using diets in which the intakes of individual nutrients are maximized in Optifood Module III).

† A ‘partial’ problem nutrient is defined as a nutrient whose requirement (i.e. 100 % of the RNI) can be met, but to the detriment of achieving the nutrient requirements of other nutrients (identified using the best diets modelled without average food patterns in Optifood Module II).

#### Selection of complementary feeding recommendations

Across all target groups, the best food sources of multiple nutrients, relative to other foods reported, were foods from the subgroups breast milk, animal milk (such as cows’ and goats’ milk), starchy plant foods (such as potatoes and green bananas) and beans, as shown in Table 3. Best sources of Fe and Zn, which were identified as absolute problem nutrients across the nine target groups, were fortified maize, black beans, potato, red meat and milk in settled and pastoralist communities, and black beans, whole grains and milk in agro-pastoralist communities (data not shown).

**Table 3:** Best sources of nutrients at the food subgroup level for each targeted age group of breast-fed infants and young children across three livelihood groups in northern Kenya, November–December 2013

		No. of nutrients* for which the subgroup was a good source†								
		Settled communities (*n* 300)			Pastoralist communities (*n* 283)			Agro-pastoralist communities (*n* 299)		
Food subgroup	Example of food item	6–8-month-old infants	9–11-month-old infants	12–23-month-old children	6–8-month-old infants	9–11-month-old infants	12–23-month-old children	6–8-month-old infants	9–11-month-old infants	12–23-month-old children
Breast milk	Breast milk	10	10	10	10	10	10	10	10	10
Fluid or powdered milk (non-fortified)	Fresh goats’ milk	8	8	7	9	8	6	5	6	7
Starchy plant foods	Potato	7	6	3	7	7	2	4	3	1
Cooked beans, lentils and peas	Red kidney beans	0	4	5	4	5	3	4	6	8
Whole grains, unenriched/unfortified	Millet flour	1	5	0	0	0	2	5	3	5
Red meat	Goat meat	1	5	3	3	1	4	4	0	0
Enriched/fortified grains, whole or refined	Fortified maize flour	6	1	2	2	2	2	0	0	0
Vitamin A-source dark green leafy vegetables	Kale	5	2	3	0	1	0	1	0	0
Vitamin C-rich vegetables	Tomato	2	1	1	1	1	2	0	0	0
Broths	Beef broth	0	0	1	0	0	0	2	2	1
Margarine (fortified)	Vitamin A-fortified margarine	1	1	1	0	2	0	0	0	0
Vitamin A-source other vegetables	Butternut squash	1	1	1	0	0	0	0	0	0
Other fruit	Mango	0	0	0	0	0	0	1	1	1
Refined grain bread, unfortified	White wheat bread	0	0	0	0	0	0	0	0	3
Eggs	Whole chicken egg	0	0	0	0	0	0	0	0	2
Vitamin C-rich fruit	Orange	0	0	0	0	0	0	0	0	1
Vegetable oil (fortified)	Vitamin A-fortified oil	0	0	0	0	0	0	0	0	1

* From a maximum of eleven micronutrients assessed in Module II of Optifood.

† A good food source is defined as a food subgroup that contributed ≥5 % of a nutrient in the diet that did not take usual observed intake into account.

#### Testing complementary feeding recommendations

The selected sets of CFR for individual livelihood and age groups are presented in Table 4. The daily consumption of animal milk and starchy foods (potatoes and green bananas) was recommended in all communities. Other commonly recommended food groups included beans across the three livelihood groups, grain products and vitamin A-fortified fats/oils in settled and pastoralist communities, and vegetables in pastoralist and agro-pastoralist communities. Fruit was included as a CFR only in settled communities, and red meat only among pastoralist communities. As expected, modelled portion sizes and frequencies were generally higher for the older than younger age groups.

**Table 4:** Complementary feeding recommendations, in addition to the recommendation to breast-feed on demand, for each targeted age group of infants and young children across three livelihood groups in northern Kenya, November–December 2013

	6–8-month-old infants		9–11-month-old infants		12–23-month-old children	
Food or food group	Servings/week	Serving size (g/d)	Servings/week	Serving size (g/d)	Servings/week	Serving size (g/d)
Settled communities (*n* 300)						
Animal milk*	14	40	14	65	21	65
Potato or green banana†	7	30	14	50	14	50
Grains & grain products‡	–	–	7	20	7	20
Beans§	7	30	7	30	7	30
Vitamin A-fortified fats/oils||	7	5	–	–	–	–
Fruit¶	–	–	–	–	7	90
Pastoralist communities (*n* 283)						
Animal milk*	–	–	14	60	21	70
Potato or green banana†	7	40	7–14	40	–	–
Grains & grain products‡	7	15	7	25	14	25
Beans§	–	–	–	–	7	25
Vitamin A-fortified fats/oils||	7	5	6	10	–	–
Vegetables††	7	15	–	–	14	15
Red meat‡‡	–	–	–	–	7	25
Agro-pastoralist communities (*n* 299)						
Animal milk*	12	40	14	40	14–21	40
Potato or green banana†	7	35	5–6	50	3–4	50
Beans§	5–6	20	7	25	7	35
Vegetables††	7	15	–	–	–	–

‘–’ means that the given food group or subgroup was not among the recommendations.

* ‘Dairy products’: cow, goat, camel and/or sheep milk.

† ‘Starchy roots & other starchy plant foods’.

‡ ‘Grains & grain products’ (with at least one fortified option): maize flour, flour mix, millet flour, rice, sorghum and/or spaghetti.

§ ‘Legumes’: pinto, red kidney, mung and/or cranberry beans.

|| ‘Vitamin A-fortified fats/oils’: vitamin A-fortified margarine.

¶ ‘Fruit’: banana, orange, papaya and/or passion fruit.

†† ‘Vegetables’: kale, spinach, carrot, tomato and/or onion.

‡‡ ‘Red meat’: goat and/or mutton meat.

As shown in Table 5, the highest number of micronutrients for which adequacy could be ensured at the population level (i.e. the nutrient’s Module III minimized nutrient content≥65 % of the RNI), using a combination of three to five CFR, was eight or nine (out of a possible eleven) in settled communities, seven to nine in pastoralist communities and five to eight in agro-pastoralist communities. Even with the best combination of CFR, the Module III minimized nutrient levels remained well below their RNI across all communities for Fe (6–34 % of the RNI) and Zn (13–47 % of the RNI). Furthermore, the Module III minimized values for vitamin B_6_ (42–51 % of the RNI) and folate (54–60 % of the RNI) remained relatively low among the older age groups from pastoralist and agro-pastoralist communities, and thiamin requirements were not met (47 % of the RNI) among agro-pastoralist communities.

**Table 5:** Summary of characteristics of complementary feeding recommendations (CFR) modelled by Optifood for each targeted age group of infants and young children across three livelihood groups in northern Kenya, November–December 2013

	Settled communities (*n* 300)			Pastoralist communities (*n* 283)			Agro-pastoralist communities (*n* 299)		
	6–8-month-old infants	9–11-month-old infants	12–23-month-old children	6–8-month-old infants	9–11-month-old infants	12–23-month-old children	6–8-month-old infants	9–11-month-old infants	12–23-month-old children
No. of nutrients that achieved≥65 % of the RNI from a maximum of eleven micronutrients for the CFR selected*	8	9	9	8	9	7	8	8	5
No. of CFR (individual food- or food subgroup-based recommendations) included in the most favourable combination	4	4	5	4	4	5	4	3	3
Minimized % of the RNI met for nutrients that remained<65 % of the RNI (%)†,‡									
Thiamin	–||	–||	–||	–||	–||	–||	–||	–||	47
Niacin§	54	–	40	51	–	41	40	35	22
Vitamin B_6_	–||	–||	–||	–||	–||	51	–||	–||	42
Folate	–||	–||	–||	–||	–||	54	–||	–||	60
Fe	6	26	–||	34	24	–||	9	9	15
Zn	18	27	30	23	20	47	13	19	23

RNI, Recommended Nutrient Intake.

* Age- and livelihood group-specific CFR selected from the Module III analyses, as detailed in Table 4.

† Defined as≥65 % of the RNI as part of a 7 d diet in which intake of the modelled nutrient has been minimized (Optifood Module III – ‘worst-case scenario’ analyses).

‡ The minimized % of the RNI was≥65 % for Ca, vitamin C, riboflavin, vitamin B_12_ and vitamin A for all target groups; as such these nutrients are not presented in the table.

§ The % of the RNI for niacin may be≥65 % and considered adequate if the contribution of tryptophan to niacin is taken into account.

|| The % of the RNI was≥65 % and the 7 d diet’s nutrient content was considered ‘adequate’; i.e. a low percentage of the population would be at risk of inadequate intake of this nutrient if this 7 d diet was consumed.

#### Consolidation of complementary feeding recommendations

Consolidated sets of CFR were compared across age groups within each livelihood group (Table 6). In settled communities, the CFR to consume daily portions of animal milks, starchy foods, grain products and beans were common across at least two of the three age groups. The complete set of four CFR could not be modelled in the 6–8-month-old settled group because it surpassed the energy allowance for complementary feeding (we modelled 67 % of energy from breast milk). The consolidated set of these four CFR across 9–23-month-old IYC was able to ensure population-level nutrient adequacy for eight of the eleven modelled nutrients compared with the nine achieved using age-specific sets of CFR. Among pastoralist communities, the age-specific CFR were more diverse and five CFR were common across at least two of the three age groups. The consolidated sets of CFR tested across the three age groups could reach the same level of nutrient adequacy as the original, age-specific sets developed for the younger age groups, but not for 12–23-month-old children. Among agro-pastoralist communities, the original age-specific sets of CFR were almost identical across age groups, with animal milks, starchy foods and beans included for all. Therefore, the consolidated set of CFR did not present any change to the number of nutrients for which≥65 % of the RNI was met in the Module III minimized diets.

**Table 6:** Complementary feeding recommendations (CFR) consolidated across age groups within each livelihood group* and the number of nutrients that achieved≥65 % of the Recommended Nutrient Intake (RNI), in the Module III ‘worst-case scenario’ analyses, from a maximum of eleven nutrients for the consolidated CFR†

	Recommended minimum servings/week		
Food or food group‡	6–8-month-old infants	9–11-month-old infants	12–23-month-old children
Settled communities (*n* 300)			
Animal milk	14	14	21
Potato or green banana	7	14	14
Grains & grain products	NF	7	7
Beans	7	7	7
No. of nutrients that achieved≥65 % of the RNI from a maximum of eleven nutrients for the consolidated CFR	7	8	8
Pastoralist communities (*n* 283)			
Animal milk	14||	14	21
Potato or green banana	7	10	10§
Grains & grain products	7	7	14
Vitamin A-fortified fats/oils§	7	6	7§
Vegetables	7	7§	14
No. of nutrients that achieved≥65 % of the RNI from a maximum of eleven nutrients for the consolidated CFR	8	9	6
Agro-pastoralist communities (*n* 299)			
Animal milk	12	14	17
Potato or green banana	7	5	3
Beans	6	7	7
No. of nutrients that achieved≥65 % of the RNI from a maximum of eleven nutrients for the consolidated CFR	8	8	5

NF, not feasible within the set constraints.

* CFR that were recommended for at least two of the three age groups within a livelihood group (i.e. food groups or subgroups with the greatest nutrient contribution) were included in the age-consolidated set of CFR with the aim to develop consistent messages across each livelihood group.

† The nutrient contribution of the age-consolidated set of CFR was tested for each age group.

‡ Foods or food groups as defined in Table 3.

§ The given food group or subgroup was not among the set of recommendations presented in Table 3.

|| The consolidated CFR could not be tested for this target group because putting them into practice would exceed the equality constraint for energy (kilocalories).

## Discussion

The current analysis demonstrates that, among 6–23-month-old IYC from northern Kenya, modifications to current feeding patterns to increase the weekly consumption of specific, nutrient-dense foods can ensure population-level dietary adequacy for five to nine of the eleven examined micronutrients, depending on the target group. Nevertheless, working within the model constraints of the current dietary patterns with regard to the types, frequency and portion sizes of foods consumed, there would still be gaps in the intake adequacy for several nutrients even if these CFR are adopted. The number of nutrients for which gaps still remained was lowest among the settled group (i.e. two or three nutrients <65 % of the RNI) and highest among agro-pastoralists (i.e. three to six nutrients<65 % of the RNI). These results are not surprising given the widespread poverty, social instability, hunger and malnutrition in these harsh arid and semi-arid regions of Kenya^(^ ^8^ ^)^.

The inability of the best combination of local foods to ensure population-level dietary adequacy for all eleven micronutrients reflects the limited number, frequency and amounts of foods that are good sources of micronutrients in the current diets and from which the modelling process could draw. For example, unlike some other settings where Optifood has been used^(^ ^19^ ^–^ ^21^ ^,^ ^29^ ^)^, the CFR for these northern Kenyan IYC did not include many nutrient-dense animal-source foods such as meat, chicken, eggs or dried fish. These foods were rarely mentioned in the 24 h recalls and availability of these foods is limited in the local food supply^(^ ^8^ ^)^. The current analysis indicates that additional modifications to the diet, well outside the current dietary patterns for these IYC, are required to achieve nutritional adequacy of the diet, particularly among agro-pastoralists. Such interventions might include fortified foods, particularly those that are fortified with Zn and Fe, or home fortification strategies (i.e. micronutrient powders). Similar results have been found, using Optifood analyses, in Indonesia and Cambodia^(^ ^19^ ^,^ ^20^ ^,^ ^29^ ^)^.

The exceptionally poor quality of complementary diets in the agro-pastoralist group was evident from the smaller number of unique foods reported as being consumed and the smaller number of foods present that are considered to be good sources of nutrients, compared with the other two communities. While there were many similarities in the selected CFR between the settled and pastoralist groups, the options for CFR among the agro-pastoralist group were substantially different and much more limited. In the settled and pastoralist groups, the addition of four to five CFR led to a progressively larger number of nutrients that reached ≥65 % of the RNI, but with little additional benefit of adding more CFR; while among the agro-pastoralists, the maximum number of nutrients ≥65 % of the RNI occurred after selecting only three CFR. This indicates that a broader range of interventions may be needed among agro-pastoralists to achieve nutritional adequacy. This finding is consistent with other studies and the FES conducted in parallel to the present study, which demonstrate that households transitioning from pastoralism to settled livelihood status often experience extreme economic hardship and food insecurity^(^ ^4^ ^,^ ^5^ ^)^.

In a similar set of studies conducted among agricultural livelihood groups in western and eastern Kenya, several of the CFR identified were distinct from these northern pastoralist populations, and included fortified porridge mixes, small dried fish, green leafy vegetables and millet^(^ ^21^ ^)^, and different intervention approaches were recommended^(^ ^30^ ^)^. These differences, that reflect variances in livelihood groups, agro-ecology, climate and culture, should be acknowledged and used to create context-relevant approaches to improving dietary adequacy in these populations.

The target population of the current analysis encompassed IYC who, in addition to undergoing the marked transition from exclusive breast-feeding to inclusion of special infant foods, to the introduction of – and the then routine consumption – of family foods^(^ ^31^ ^)^, were from three very distinct livelihood groups in northern Kenya (i.e. nine target groups). Optifood’s approach of developing context-specific recommendations for individual target populations means that analysis occurs in isolation for each age-based target group (due to differences in food patterns and RNI) and, typically, Optifood projects have focused on only one or two target groups simultaneously^(^ ^19^ ^,^ ^20^ ^,^ ^32^ ^,^ ^33^ ^)^. The development of common CFR across the age groups in a population, as was done in the present study, would facilitate their promotion and adoption by expecting caregivers to adopt and maintain a single set of practices for the duration of the complementary feeding period as opposed to recommending new foods or practices at the 6-, 8- and 12-month marks. Unlike previous Optifood studies^(^ ^19^ ^–^ ^21^ ^,^ ^32^ ^,^ ^33^ ^)^, we used a structured methodology in which we selected CFR with a high nutrition impact across the majority of age groups and applied a consolidated set of CFR across all age groups. Age-specific portion sizes and frequency of consumption were maintained to reflect the natural progression towards a larger energy contribution from complementary foods as children get older. The limitations of the CFR consolidation method used here are that children at either the younger or older end of the complementary feeding period may be disadvantaged; and the approach should not necessarily be used in populations where infants <1year old are fed special foods, such as fortified infant porridges. An alternative approach may have been to take CFR that had been identified for only one age group and test them for the remaining two age groups in that livelihood area. The disadvantage in this method would be a greater number of resulting CFR or recommended behaviours, which in practice may be difficult to promote.

A further step towards improving dietary adequacy, which was not done in the current analysis, would be to add and test foods that were not consumed by a specific age group, but were consumed by other age groups within the same area. On the one hand, this approach could lead to CFR that include more nutrient-dense foods available to the population; on the other hand, it could potentially ignore the nuanced evolution of children’s diets in the study areas and resulting CFR could prove unsustainable. Further consolidation of CFR may be considered across seasons or in different geographical regions. In the present study, even though consolidated messages would not achieve the same potential improvements to IYC nutrition, the expected benefit is likely still substantial. Although, mathematically speaking, age-specific CFR would achieve the highest nutrient potential, in practice, simpler, streamlined messages for all young children are easier to implement as part of a behaviour change communication strategy^(^ ^34^ ^)^ and potentially more likely to be adopted.

The analysis using Optifood presented herein provides technical information regarding CFR that could ensure improved dietary adequacy for most individuals in the target groups. A critical next step is to examine the context-specific feasibility, acceptability and affordability of implementing the selected CFR to design a realistic behaviour change communication intervention^(^ ^14^ ^,^ ^19^ ^,^ ^20^ ^,^ ^35^ ^)^ and develop further strategies beyond behaviour change communication that are needed to bridge nutrient gaps in the local food supply, particularly important in this context of significant poverty and food insecurity. It is critical to bring the results of the Optifood analysis and FES together to identify opportunities and constraints in applying the CFR, including perceptions of cost, convenience, accessibility and appropriateness of the recommended foods for IYC diets and other social or physical factors that determine accessibility of these foods across seasons^(^ ^30^ ^)^. This will inform whether the selected foods and their quantities are suitable for promotion in the represented populations, or whether they need to be adapted or substituted to facilitate adoption. There are several factors that may affect adoption of CFR in practice; these include availability of foods on-farm or in the market across different seasons, their cost as well as affordability based on the local economy, food preparation time, individual child preferences for foods, and current beliefs and practices. Food shortage is prevalent among these populations; overall, one-third participated in a food programme within the last year. The cross-sectional dietary survey used as the basis for the Optifood analysis did not collect information on the sources of foods reported in the 24 h recall. Results of the FES conducted in parallel to the study presented herein showed that many of the forty-eight FES respondents in Turkana received donations of sorghum (*n* 25), beans/lentils/split peas (*n* 13) and oil (*n* 12)^(^ ^8^ ^)^. Food donations were less prevalent in Isiolo and Marsabit, with respectively ten of forty-eight and seven of thirty-six FES respondents reporting having received food. It is recommended that testing of the feasibility and acceptability of these CFR be carried out using tools such as ProPAN^(^ ^36^ ^)^ or Trials of Improved Practices^(^ ^37^ ^,^ ^38^ ^)^ where caregivers are asked to trial and provide feedback on the CFR.

A series of limitations inherent to dietary and Optifood analysis is acknowledged. The analysis is dependent on the quality of the dietary recall data, estimated breast milk intakes, the food composition data and RNI used. As data collection took place during the rainy season, the results cannot necessarily be extrapolated to other seasons. It was not within the scope of the study to collect data across all seasons and develop season-specific CFR. Further, while particular foods appeared in the list of foods consumed by the target population, these foods may not be accessible to all local families or at all times. With respect to the RNI, the Zn requirement proposed by the WHO/FAO is higher than that proposed by the International Zinc Nutrition Consultative Group^(^ ^39^ ^)^. Although phytate intake was not directly taken into account, intake was assumed to be relatively high based on the diet type and the bioavailability of Zn and Fe was assumed to be low. Similarly, Vitta and Dewey^(^ ^40^ ^)^ argue that the WHO/FAO RNI for Ca may be inflated and therefore propose use of the more recent US Institute of Medicine Dietary Reference Intake for analysis of Ca intake adequacy^(^ ^41^ ^)^. The estimation of niacin inadequacy is limited by the lack of information on tryptophan conversion. For breast milk, the estimated energy contributions from the proposed CFR were systematically lower than the observed energy intakes from complementary foods, which suggests that the surveyed children may be consuming less breast milk than modelled. Breast milk was an important food source for ten of the eleven nutrients modelled (Table 3); therefore, differences in the modelled and actual breast milk intakes could modify our findings.

In summary, the present study highlights the potential value and limitations in determining age- and population-specific CFR that take into account the actual, local dietary patterns and available foods. Localized CFR can provide much more specific and nutritionally relevant recommendations than general IYC feeding guidelines. However, the complexity and specificity of localized CFR which aim to simultaneously meet multiple nutrient requirements need to be balanced with the practicalities of feeding IYC and comprehensibility of behaviour change messages. We found trade-offs in the nutrient adequacy of the CFR as a result of simplifying and consolidating CFR across age groups.
